# Expert perspectives on priorities for supporting health security in the Pacific region through health systems strengthening

**DOI:** 10.1371/journal.pgph.0000529

**Published:** 2022-09-22

**Authors:** Nicole Rendell, Meru Sheel

**Affiliations:** 1 National Centre for Epidemiology and Population Health, ANU College of Health and Medicine, The Australian National University, Canberra, ACT, Australia; 2 Sydney School of Public Health, Faculty of Medicine and Health, The University of Sydney, Camperdown, NSW, Australia; Public Health Foundation of India, INDIA

## Abstract

The COVID-19 pandemic has reiterated the interdependence of health security and health systems, and the need for resilient health systems to prevent large-scale impacts of infectious disease outbreaks and other acute public health events. Three years into the COVID-19 pandemic has led to discussions on how to “build back better”, making it important to identify lessons to strengthen health systems and prevent future shocks from health security threats. Limited data exist on effective implementable initiatives, especially for the Pacific region. We explored the perceptions of a selection of experts with field experience in the Pacific region to identify and prioritise areas for future health system investments that strengthen health security. We conducted a qualitative cross-sectional study, collecting data using four focus group discussions. We analysed the data using a content analysis of notes recorded from each of the sessions. There were 24 participants, representing 15 research and academic institutions, nongovernment agencies, UN agencies and government as well as independent consultants. All were health sector stakeholders with field experience in the Pacific region and expertise in either health systems or health security. The analysis revealed four areas to prioritise future efforts, namely workforce development, risk communication, public health surveillance and laboratory capacity. A fifth theme, localisation, was identified as a cross cutting theme that should be applied to implementation of other identified priority areas. These findings provide a starting point to apply in practice this relatively new concept, of targeted health systems strengthening for health security development, in the Pacific. Evaluation of these initiatives will strengthen knowledge on the value of integrating these two concepts.

## Introduction

The interdependent relationship between health systems and health security is long known. However, it is not well understood and there are few examples of purposively integrating the concepts into practice. The COVID-19 pandemic has highlighted the role of health systems in promoting and strengthening health security. This has prompted a global re-think on how we approach initiatives to promote health security, with a renewed focus on strengthening health systems and considering health security in the context of resilient health systems. As we “build back better” [1], it is important to identify lessons following the impact of the COVID-19 pandemic, including adopting a health systems approach to determine the priorities for health system and health security initiatives in ways that they mutually reinforce each other.

Health security is defined as “*the avoidance and containment of infectious disease threats with the potential to cause social and economic harms on a national*, *regional or global scale*” (page ix) [2] The COVID-19 pandemic has encouraged policy-makers, practitioners and researchers to move beyond implementation of the International Health Regulations (IHR) (2005) [2–5], and reconsider additional approaches to health security through strengthening health systems in alignment with the IHR 2005 [6]. This revised approach to health security acknowledges its interdependent relationship with health systems and aspects of health security that were not always directly encompassed by the IHR (2005). In doing so, we can contribute to the development of more resilient health systems that are better equipped to prevent direct and in-direct impacts of acute public health events like disease outbreaks [7].

Multiple frameworks and definitions exist for both health systems and health security however only recently have these concepts been merged into a single framework [7, 8]. We adopted the World Health Organization’s (WHO’s) definition described in the context of health security in the 2021 Health Systems for Health Security framework which suggests:

“*Health Systems for Health Security (HSforHS) is an approach that harmoniously brings together efforts to strengthen resources and capacities required for implementation of the International Health Regulations*, *components in health systems and those in other sectors for effective management of health emergencies*, *while maintaining the continuity of essential health services throughout*” [7].

This *HSforHS* framework extends beyond IHRs (2005) implementation and incorporates the dynamic role of factors inside and outside of the health system. For example, during acute public health events, response efforts may need to be coordinated with government departments responsible for defence and the environment in addition to the relevant health department. Establishing mechanisms within the health system to promote collaboration among these departments will support a more effective response. The concept for *HSforHS* is further supported by other global initiatives in response to the pandemic such as the Independent Panel for Pandemic Preparedness and Response [9, 10] and evidence from implementation of effective national responses [11].

Evidence and practical examples of health security development through health system strengthening (or vice versa) in the Pacific region are limited, and not many of them have been evaluated. A 2019 report on health security based on data from the Region (the State of the Region 2019 report), identified areas for health system strengthening to promote health security that align with the *HSforHS* framework [2, 7]. The report recommended strengthening health security in the region by improving capacity to detect and respond to outbreaks of zoonotic diseases, increasing antimicrobial stewardship activities, improving laboratory capacity, maintaining immunisation coverage and implementing workforce initiatives that can facilitate a deployable highly skilled workforce during emergencies. The report also recommended improving real-time surveillance and information management systems, both by design and day-to-day application, as well as integration across sectors and between countries [2].

More recently, a desk review by Linhart et al (2021), on health system strengthening in selected Asia-Pacific countries (Cambodia, Fiji, Indonesia, Laos, Papua New Guinea, Solomon Islands and Vietnam), identified key challenges and opportunities in the Asia-Pacific region [12]. While focussed on health systems, the review recommended investments that overlap with key aspects of health security including human resources for health, health service delivery and health information systems. Specifically these recommendations advocated for improving the quantity and quality of the workforce with targeted strategies for rural and primary health care workforce; improving the quality of community health services; increasing preventive and population health interventions; long term investment in health information systems infrastructure and workforce; strengthening data quality and data culture; and increasing private sector health information reporting compliance [12].

Health systems of countries within the Pacific region, and specifically the small island developing states of the Pacific, experience additional challenges. These include delivery of and access to health services to small geographically dispersed populations with limited transport links and connectivity options [13]; the increasing burden of non-communicable diseases especially diabetes and obesity, [14, 15]; chronic workforce shortages; weak governance arrangements; supply chain and logistical issues associated with island nations and limited information systems [16].

In the Pacific region, strong border controls implemented during the COVID-19 pandemic, limited the direct impact of COVID-19 morbidity and mortality in 2020 and 2021. For the first two years of the pandemic, some of the few countries in the world that had not reported any local transmission of SARS-CoV-2 were in the Region. However, during this time the Region experienced multiple in-direct effects of COVID-19, including fiscal losses from border closures and disruption to tourism; issues of food security; increased reports of mental health issues including anxiety and depression; disruption of routine health services particularly routine immunisation and maternal child health, and disruption to workforce learning and development [17, 18]. However, with the emergence of the Delta and Omicron variants of SARS-CoV-2, widespread community transmission has reached all but two of the Pacific Islands and Territories, as of July 2022 [19, 20].

As we emerge from the acute phase of the COVID-19 pandemic response, it is widely acknowledged that there will be opportunities to strengthen health systems, in a way that promotes health security, which must not be missed [7]. In light of this, we aimed to identify health system strengthening initiatives to prioritise, recognising the lessons learnt from the COVID-19 pandemic, with focus on those initiatives that prevent impacts of and strengthen the ability to respond to health security threats in the Region. In this paper we aimed to identify priorities to improve health security through implementable health system strengthening initiatives in the Pacific Region.

## Materials and methods

### Study setting

For the purposes of this study, focus countries referred to collectively as the Region, include Timor Leste, Papua New Guinea and Pacific Island Countries and Areas, here after collectively referred to as the Region. Pacific Island Countries and Areas include the Cook Islands, Fiji, Kiribati, Republic of Marshall Islands, Federated States of Micronesia, Nauru, Niue, Palau, Samoa, Solomon Islands, the Kingdom of Tonga, Tuvalu and Vanuatu.

### Study design

We used an exploratory qualitative cross-sectional research design [21]. We used online focus group discussion (FGDs), where the objective of each session was to engage stakeholders with relevant experience in the Region and obtain their views on the current challenges in health security facing the Region, how these could be avoided or mitigated using initiatives that target strengthening health systems, which initiatives should be prioritised and how these should be implemented. Key informants had expertise in public health, health systems, health security and/or international development in the Region.

We purposively sampled participants using study researchers’ networks to ensure all participants had health security or health system expertise, as well as field experience in at least one country within the Region. We contacted 39 individuals by email and invited them to participate in the FGDs. Participants represented research and academic institutions, nongovernment organisations (NGOs), UN agencies and government as well as independent consultants. A list of all representative organisations is presented in S1 File.

### Data collection and analyses

We conducted four online FGDs in November 2021. Prior to each session, participants were provided with an information sheet, for consent purposes, and a participant guide containing important context and definitions (S2 File). Each participant was required to provide their oral informed consent at the beginning of each session before proceeding. We used a semi-structured facilitation guide to support each session (S3 File). This guide was developed based on gaps in health security identified in the literature and proposed the following ideas for consideration: surveillance, laboratory capacity, health workforce, immunisation, primary health care/community health, risk communication and antimicrobial resistance. Open questions were also added to explore possible additional themes and explored topics such as key strengths, challenges, strategies to mitigate or avoid identified challenges, priorities and approaches to implementation.

The FGD sessions were conducted using video conferencing software. Both study authors were present for all FGDs. MS, an experienced epidemiologist, interviewer and facilitator who has extensive field experience in the Region, facilitated the FGDs. NR, a health systems researcher whose ongoing PhD candidature research in based on countries in the Region, used a note-taking method to document the discussion from each session, which were then verified against the recording before it was deleted. Inductive content analysis of the notes was undertaken (NR) to identify key themes and agreement was reached by consensus.

### Ethics

This study was approved by the Science and Medical Chair of the Australian National University Human Research Ethics Committee 26 October 2021, Protocol 2021/656.

## Results

We conducted four FGDs with researchers, practitioners and policy makers working in public health, health systems, health security and international development that all had experience working in the Region. Of 39 invited experts, 24 participants (62% response rate) from 15 different organisations (see S1 File for list of associated organisations) attended the FGDs. For most of those that did not participate, the reason was scheduling conflicts. All participants had experience in the countries of interest, primarily Fiji and Papua New Guinea, with different levels of professional and field experience ranging from 3 years to 35 years. The first three FGDs used the same facilitation guide. The guide was updated for the last FGD because data saturation on key challenges was reached, and we wanted to explore further priorities for action in response to previously identified challenges. The duration of each of the FGDs was around one and a half hours.

Through content analysis of the FGD notes, we identified five main areas to target health system strengthening initiatives that will contribute to promoting health security in the Region (see S4 File for a full summary of extracted themes and supporting quotes):

- Workforce development
- Risk communication
- Public health surveillance
- Laboratory capacity
- Localisation.

Additional points of interest were raised during the FGDs which warrant reporting however they were not considered dominant themes. These are reported below as *Other emergent topics*.

### Workforce development

Workforce shortages emerged as a dominant theme across all discussion groups and were considered by most participants to be the key component of the health system that needed to be strengthened in the Region. All groups were clear that the quantity workforce available in the Region is limited and investments were needed to focus on training new health care professionals and consolidating the available workforce. In addition to training new health care professionals, it was also seen as important to strengthen their ongoing capability and capacity.

*“Workforce capacity building is constantly neglected in the Pacific*. *We know from the research and the data that there just isn’t enough population or people to be able to get the numbers that we need in terms of health care professionals*.*”* FGD#2

“… *the workforce capacity is not sufficient for day-to-day operations let alone surge capacity… Need to address support for existing health professionals*” FGD#2

Consideration of skill mix or task shifting were proposed as ideas to manage health workforce shortages. This includes supporting those health care workers with a broad skillset, in geographically isolated areas, to service the full needs of their community, and providing continuing professional development options.

*“…major opportunity was primary health care strengthening and a recognition that most frontline care does not require physicians”* FGD#2

*“The concept of task shifting*, *building up capacity at the community level*, *that’s where we’ve seen success*.*”* FGD#2

*“The more eager and hyper segmented our assistance becomes*, *the more it runs counter to sensible health workforce development…From a health security perspective and what workforce means*, *it actually means a generalised health care workforce with some public health capacities*.*”* FGD#4

It was noted repeatedly that the “fly in, fly out” model from Australia was not working in its current structure (for example, AUSMAT [Australian Medical Assistance Teams] as a deploying mechanism). In three of the four FGDs, participants expressed strong opinions on the need to move away from this model or reserve it for very specific circumstances. In one FGD, experts suggested that the model could be strengthened to deepen its impact and provide greater capacity for public health response rather than emergency medicine. All participants agreed that there was a place and an opportunity for public health emergency surge capacity to be deployed from Australia but also from within the Region.

“*Countries like Australia have interest in fly in style but should never replace national rapid response*. *Sometimes necessary where there’s an acute surge*. *Coupled with long term capacity building*.” FGD#3

“*We already had the view pre pandemic that there was a case for deepening the pool and broadening the pool of deployable public health expertise*.” FGD#4

Additionally, some FGD participants noted the overlap between building capacity and digital capability. Travel restrictions during the COVID-19 pandemic have enhanced the use of digital tools and increased reliance on internet connections. This was the only means during the pandemic to connect NGO partners with relevant staff in-country. While there is still a need for face-to-face meetings, training and partnerships, especially in the context of emergencies, participants agreed there is an opportunity to embrace digital solutions that are locally led on the ground. Investments in digital infrastructure have the potential to continue this momentum, promote autonomy and develop national workforce.

*“What we’ve learnt is there’s a need to invest in online facilities*, *in terms of primary health care training or advanced surgical training for experienced practitioners… there’s a real need to look at ways [to]… invests in that sort of online capacity*. *Infrastructure training*, *modules*, *digital platforms*.*”* FGD#2

### Risk communication

Risk communication emerged as a strong theme in two of the four FGDs, especially among those working within or closely with NGO partners, or who had strong connections with those working “on the ground” in the Region. Repeatedly, the concerns raised around risk communication were discussed in the context of vaccination, largely prompted by issues around the rollout of COVID-19 vaccination programs–a widely known challenge in Papua New Guinea. Prior examples of the 2018 polio outbreak in Papua New Guinea and measles outbreaks in Papua New Guinea (2018) and Samoa (2019–2020) were raised by FGD participants as examples where communication hurdles had been experienced. Interestingly, risk communication was not raised as an issue by those affiliated with government agencies and when prompted, their concerns were more around governance and finance.

“*This extends beyond the general public to the communication of advice that’s being given to government including the level of urgency applied to that advice*.” FGD#1

Any resources available to promote evidence-based messaging were viewed positively, but most FGD participants agreed that these were only useful to countries and communities if there were resources on the ground that could contextualise (for local language and culturally) such resources to the specific setting.

“*Risk communication is an important part of any response*. *Just communication resources that have been produced elsewhere are rarely appropriate for the current country*. *Need capacity in place to identify need to rapidly adapt*. *They can then have resources made locally appropriate*, *field tested and rolled out*.*”* FGD#3

When discussing possible initiatives that improve risk communication, the platforms used for communication and issues around counter-messaging misinformation were both highlighted.

*“Restoring funding to the ABC [Australian Broadcasting Corporation] Pacific Australia network…There are many communities where radio is still the lifeline to the outside world*. *Having access to that network again*, *they would be able to hear very informed*, *clear public health information*.*”* FGD#1

“*Facebook [is the] only source of information in PNG [Papua New Guinea] at the moment and it is full of rumour and misinformation*. *Our programs are on Facebook trying to provide information but not using artificial intelligence*. *It’s very challenging to counter misinformation*.” FGD#1

### Public health surveillance

Issues around surveillance were consistently raised across all FGDs. However, there were mixed perceptions as to what degree surveillance should be prioritised in the forward agenda for the Region. One of the main surveillance-related concerns raised by FGD participants was in the context of data use for informed decision-making and operational research.

“*There was a lost opportunity or intent in the capacity and capability for research in line with the pandemic*, *and the crisis going on to give us real time data that is good for policy and decision-making*” FGD#3

“*There’s information that gets gathered but countries being able to really make use of that and use it to drive their priorities*. *It’s a long term project*.” FGD#4

Another component of surveillance that was discussed in the FGDs was the lack of data on vulnerable populations.

“*It all starts with data*, *so having that disaggregated disability*, *gender*, *age*, *disaggregated data in the very beginning and how much that could help the response because responses are so urgent and immediate*.”FGD#1

“*Only now have time [to] consider [the] pattern of distribution*.”FGD#4

A few FGD participants raised the issue of expanded digital technologies and electronic apps being used for surveillance, monitoring disease trends and case detection; however, the data did not always lead to public health action or aid decision-making, a critical component of surveillance.

*“One of the key elements to decentralisation is health records and access to them. There are some e-records at the central level but [but mostly] still using paper at community level. Need to move online as much as possible but need to ensure access at community level [which is] very difficult.” FGD#2*

Ideas to improve use of surveillance data were discussed briefly. One idea suggested was the role of advocacy or operational research champions. Another was an extension of the ideas raised under workforce development, where the focus is on ensuring the appropriate people inside and outside the health sector are familiar with the tools and processes ahead of an outbreak situation as part of preparedness.

*“We need to support identification of local champions for advocacy research*. *These are the people that can work with the data*. *Identify operational research priorities… They tend to work in public health but not in government*. *Senior and central*. *Tends to be academics at mid-level institutions*. *These champions can promote critical thinking and drive local responses [as] trusted technical partners*.” FGD#2

“*Teams will come in with surveillance tools which can be great for a short time*, *but they’re not really building national capacity*, *so having the tools and the people trained on those tools ahead of time is very important to be able to exercise those capacities with tabletop exercises*, *simulations*, *and having the people not just from health*.” FGD#3

### Laboratory capacity

Concerns around capacity of national laboratories and their staff did not emerge as a strong theme in the FGDs. However, it was raised as a priority in one FGD among those participants based in the Pacific and received enough attention in the other FGDs to warrant its inclusion as a priority area for consideration. Most participants felt that strengthening laboratories, the capacity of the workforce and their networks had historically received little attention, and before the COVID-19 pandemic had largely been neglected. However, as a result of investments in laboratories that were made in response to the pandemic, laboratory facilities in the Pacific have improved dramatically. The participants did raise concerns though about the sustainability of these investments.

“*Lab strengthening … neglected for a long while*.” FGD#4

“*Laboratory systems have improved ahead of pace but covering the cost of consumables is an ongoing factor*.” FGD#3

“*One of the key issues with the emergency response was introducing new things rather improving existing things*. *For example*, *lab capacity was improved with surge in supplies of GeneXpert machines to support COVID-19 testing*, *but the systems in place have not been able to budget for their ongoing costs*.” FGD#3

### Localisation

This theme emerged consistently across all FGDs as a model of practice in the context of implementation, and as such, can be considered as a cross cutting theme that applies to all other identified priorities. Although no FGD was able to identify a specific example, it was clear that partnerships and coordination with local community organisations were perceived as essential to the success of any initiative.

“*Tailoring response and development strategies to countries–usually one size fits all applied to Pacific but each country has its own specific context that we need to take into account so it is better to tailor*.” FGD#1

“*Local empowerment… Institutional linkages are one [of] the most effective mechanisms… Partnerships between academic institutions are peripheral*, *NGOs vary but it’s not them either*, *it’s community organisations*.” FGD#4

Extending on the above concept of engagement with community level organisations, some participants highlighted further the nuances of collaborating with local organisations. They emphasised that the ideal partnership is one that is purpose built to achieve the intended outcome.

“*Depends on issues–sometimes need more complex relationship*. *Much more nuanced*. *As local as possible in first instance but may need tailored support depending on what you’re looking at*.” FGD#1

“*We need to consider who are the critical stakeholders in the Pacific*. *Our organisation has had success collaborating with faith leaders*. *This approach uses what’s already trusted faith networks and civil society*.” FGD#1

The FGD participants also suggested some reasons as to why implemented initiatives in the Region have failed in the past. These centred on an absence of engagement with the right stakeholder locally, or a structural mechanism that limits the opportunities for such arrangements to exist.

“*There is a problem with over-piloting*. *Attempts to scale tend to fail because they are not developed with [the] right partners from the outset*.” FGD#2

### Other emergent topics

Several additional interesting topics and themes emerged through the FGDs which merit presentation:

- Disruption in routine immunisation during the COVID-19 pandemic highlighted difficulties in maintaining progress of immunisation programs other than for COVID-19.

*“Difficulties have emerged for the broader expanded program on immunisation. While we see a rapid acceleration of our first and then second dose coverage [of COVID-19 vaccines] across the Pacific, data shows routine immunisation coverage, measles and rubella coverage for example, may have slipped as low as 50% coverage in a number of countries.” FGD#3*

- Wellbeing of populations and the health workforce was negatively impacted by COVID-19 measures.

*“Generally wellbeing is not addressed well in the health security preparations when the pandemic is quite lengthy. For example, health workforce and general population through lockdowns.” FGD#3*

- Maintaining primary health care and community services during crises is vital but difficult.

*“Evidence shows more people died from maternal causes than Ebola [2014 outbreak in West Africa]. When there is a crisis, we should be allocating funding to what’s causing the most death and disability accordingly rather than focusing on what’s the ‘new thing’.”FGD#1*

- One regional expert noted the contribution of and learnings on infection prevention and control measures in the Pacific, especially in the context of Fiji.

*“So if this pandemic was a measure of how good a system was intended, IPC [infection prevention and control], it exposed the system and we really need to do a lot of work in this area.” FGD#3*

- While gender and disability did not appear as themes in the FGDs, some participants highlighted gaps in data on gender and people with disability. One participant highlighted that the pandemic for the first time saw the inclusion of people with disabilities at “decision-making tables”

“Both the Australian Humanitarian Partnership and Pacific Disaster Ready, set up in PNG [Papua New Guinea], Solomon Islands, Fiji and Timor Leste, are examples that have improved capacity among local disability organisations to advocate for disability inclusion. In terms of COVID-19, this means more people at the table in planning the response [that consider disability inclusion].” FGD#1

## Discussion

This study explored the perceptions of relevant health system and health security experts on the priorities for health system strengthening to contribute to the development of health security in the Region, following the peak of the acute phase of the COVID-19 pandemic. Collectively, the four FGDs identified four key priorities: workforce development, risk communication, public health surveillance and laboratory capacity. A fifth theme, localisation, was also identified as a cross cutting theme and functionally applies to all other identified priorities.

The FGD findings on workforce development align with recent literature that recommends investing in strengthening the capacity of the health workforce of the Pacific, specifically community health workers [2, 12, 22]. In 2019, WHO reported the latest available data on density of doctors per 10,000 population by country. Densities for countries in the Region 8.6 for Fiji (2015), 5.4 for Tonga (2013), 3.5 for Samoa (2016), 0.66 for Papua New Guinea (2019) and 7.7 for Timor Leste (2019). These were much lower than for Australia (37.6; 2018) and New Zealand (34.2; 2018) [23].

The majority of doctors in the Region are concentrated in tertiary hospitals, with institutions unable to train and deploy sufficient numbers of clinicians [24]. Only two institutions in the Region (one in Fiji and one in Papua New Guinea) provide post-graduate training. The distribution of the workforce is also unequal, with many outer islands having no permanent medical staff at health facilities for many years [24]. Health districts are frequently inadequately staffed [25].

There is some evidence on the use of digital tools and innovation for health workforce development. The pandemic has also highlighted the role of digital health interventions that can support the development of health workers by providing training and supervision, and facilitating communication. Evidence from low- and middle-income countries demonstrates that implementing digital tools is an effective approach in small-scale interventions and pilots [26]. However, how best to guide design or implementation on a larger scale is unclear, as the evidence quality in support of such programs is variable and cannot be readily generalised [26–28]. This means a cautious and deliberate approach to digitally based systems and workforce development initiatives is needed, to ensure all external, system-level, institutional, ethical, safety and individual factors are incorporated by design prior to implementation [27, 28]. For example, in the case of the Pacific region, consideration would need to be given to the infrastructure requirements and physical capacity for connectivity in geographical remote areas, which is an access issue independent of the pandemic.

Our FGDs showed that there is an issue with effective risk communication in the Region that was exposed during the COVID-19 pandemic. Risk communication during outbreaks, acute public health events and vaccination campaigns is essential to promote trust in public health recommendations. Several lessons around the importance of effective risk communication arose from the 2009 H1N1 “swine flu” pandemic but came to the forefront during the West Africa Ebola outbreak (2016), the Pacific measles outbreak in Samoa (2019) and also the COVID-19 pandemic [29–32].The emergence of social media as a platform for wide-scale and rapid information sharing was useful and advantageous for rapidly disseminating information and encouraging community participation, but was accompanied by the emergence of the mis-information “infodemic” [33, 34]. The COVID-19 pandemic further exposed this vulnerability in the Pacific [35]. Increasingly, the public health field needs to build mechanisms to tackle mis-information in real time, through engagement of local communities and government stakeholders. In the Pacific, it has been recommended to target strengthening official credibility and visibility online, encourage collaboration among stakeholders in their approaches to tackle misinformation and prepare for new and emerging threats online that undermine credible information sources [35].

A recent rapid scoping review sought to synthesise evidence on the different modes of health risk communication to the public from the H1N1 and COVID-19 pandemics [36]. The review identified an overall lack of experimental studies examining the link between self-protective behaviours and the use of social media by health authorities. The authors recommended that more studies are needed across the fields of both health risk communication and media studies. Similar outcomes were reported from the first ever global infodemiology conference, organised by WHO. Participants advocated for public health authorities to develop, validate, implement, and adapt tools and interventions for managing infodemics in acute public health events in ways that are appropriate for their countries and context [37, 38].

Public health surveillance was raised by the FGDs as a key area for future investment but primarily in the context of using data use for decision making and operational research. Capacity to detect infectious disease cases and events is an essential function of health systems and, critical for early detection of outbreaks and responding to health security threats. Capacity for detection includes surveillance, health information systems, laboratory diagnostic capability, and epidemiological expertise to transform surveillance data into useful, actionable intelligence to inform decisions and policies [2]. However, weak surveillance systems including limited maturity of multi-source surveillance, inconsistent case definitions, and lack of awareness among clinicians and public health officials contribute to underreporting and delayed public health response.

The need to strengthen public health surveillance initiatives are well recognised but like the data presented by our study, the recommendations tend to be nuanced. The review by Linhart et al of the health system needs in the Asia-Pacific region, prioritised data quality and data culture aspects of surveillance [12]. These included regular feedback to subnational levels of the health system about the quality of data being collected, and how data are being used to guide decision-making. Multiple studies have reported that regular feedback to the sub-national and health centre level is currently lacking in most countries due to a lack of human resources available to provide this feedback, and a lack of established mechanisms to efficiently disseminate it [12, 39]. While there has been an increased momentum and advocacy for more specifically for real time surveillance [2], focussing on system integration across different levels of the health system and greater use of data for decision-making are also needed.

Improving data use through a network of “champions” is one measure advocated by Measure Evaluation (an NGO affiliated with USAID) in their extensive work on data demand and use [40, 41]. Champions are usually motivated employees who receive targeted training to support and advocate data use activities within their districts [40]. However, the effectiveness of this strategy remains unclear. In practice, the needs of individual systems, and the application of those systems in different communities, will vary across the Pacific.

Another approach raised in the literature, though not directly identified through the FGDs, is the use of health-facility-based event-based surveillance. Previously championed and implemented in some of the Pacific island nations, this approach holds potential for early detection of health security threats in settings with limited resources, and closely models principles of community-based and syndromic surveillance [39, 42].

Strengthening laboratory capacity was identified as a priority for the Region and offers another example of the value of aligning health security and health system perspectives. Although work was already underway to strengthen laboratory capacity in the Region prior to the pandemic, the 2019–2020 Pacific measles outbreak found that shipment of specimens from the Pacific islands to Australian laboratories, led to delays in detection and response to outbreaks in some contexts, and presented an opportunity to strengthen the response to future outbreaks [31]. Pandemic preparedness and response in the Region has accelerated laboratory strengthening in the Region, with establishment of mobile molecular laboratories, establishment of RT-PCR testing equipment (GeneXpert) and provision of virtual training [43].

Multiple Australian training programs and NGOs are working towards meeting the current need for workforce strengthening in the Region. For example of the Pacific Regional Infectious Disease Association (PRIDA), an Australian based network that offers training on advanced testing capacity, infection control and antimicrobial stewardship [44]. However, these programs rely on funding and partnerships through other larger organisations.

Moving forward, sustainable funding for ongoing costs of laboratory infrastructure, equipment maintenance and workforce training (testing and data analysis), along with expansion and integration of laboratory surveillance for other health security threats such as tuberculosis(TB), malaria, vaccine-preventable diseases and emerging infectious diseases, will be important considerations.

The most consistent theme throughout all the FGDs was the integral role of localisation. Engaging with local civil society is also repeatedly highlighted in the literature but no specific recommendations have been presented in recent evaluations that target regional recommendations [2, 12]. As well as being a moral imperative, evidence suggests that community engagement more broadly is an effective way to attain positive health outcomes. It is the evidence on how to approach that engagement that is lacking [45, 46]. This point was further reiterated in the references to the ‘fly in fly out’ approach that was discussed in context of workforce development.

An important consideration to effectively engage local organisations is the role of decolonisation. While not explicitly raised by the FGD participants, much of the discussion was based on the role of international NGOs and Australian based organisation on supporting health security the Pacific. A colonial history in the Region means that systems of dominance and power are deeply ingrained into the systems within which we work today, and, realistically, any effort for collaboration needs to take measures to counter the impact of such systems [47, 48]. For example, one barrier to establishing effective working partnerships between Australia and countries in the Region is that such partnerships can create power differentials that mirror colonial relationships. A best practice implementation approach to decolonise partnerships remains ill-defined [47, 49]. However, some approaches for moving forward have been proposed in the literature. One approach is for global health practitioners from high-income countries to immerse themselves in the communities they serve, for situations and settings where it is identified by the community that assistance is needed, to minimise power differentials and allow close communication between all parties, especially around establishing frameworks to resolve ethical challenges [49]. Recently, a comprehensive and pragmatic *Equity Focused Tool for Valuing Global Health Partnerships* was published and holds potential for supporting open dialogue in these partnerships. The tool is structured as a simple traffic light rating across key criteria in four domains (Governance & Process; Procedures & Operations; Progress & Impacts; and Power & Inclusion) and is designed to prompt reflective dialogue among all parties within the partnership [48].

In addition to local NGO partnerships, there is a greater need to leverage academic institution partnerships to guide local research and context-specific evidence-based decision-making [47]. The imperative is even greater in the Region, where there are only few education and research institutions; enhancing their leadership in the Region will help mitigate future knowledge and workforce shortages. Moving forward will require models that enable more collaborative framing that resists mainstream use of “local” actors within a “traditional” system [50].

Using these findings as a primer to identify priority areas for health system strengthening and health security, there are several recommendations for policy makers and nongovernment organisations to apply in practice. Each of the themes represent an area of health system strengthening, that contributes to health security, that could be prioritised when planning immediate and medium-long term investments in the Region, in consultation with local stakeholders. Recommended activities include:

- Localisation should be considered the universal model of practice and apply to all activities, programs and initiatives, acknowledging the role of decolonisation.
- While workforce needs were highlighted, prior to new programs, it will be important to conduct a review of the workforce with a focus on the public health workforce. This would facilitate an understanding of the quantity and quality of workforce engaged in the COVID-19 pandemic would provide welcome insight on how to manage the identified workforce shortages. The review should include what roles were filled, what staff were doing, how they acquired the skills and who missed out. Doing so will allow consolidation of the workforce and identify surge mechanisms for the future.
- Apply social and behavioural economics to design future risk communication strategies and build and integrate formal networks of community mobilisers trained in risk communication who can adapt and use culturally appropriate tools. Such training could occur over the medium-long term and include in the principles of health promotion, risk communication, management of misinformation and anthropology.
- Invest in strategies that promote use of data for decision- making, with a focus on using surveillance data, closing feedback loops and using local data to guide responses. Additionally, use local evidence and support local research through local and regional academic and research institutions. This could be achieved by creating regional-based knowledge translation hubs, such as subregional technical advisory groups for different areas, to allow cross-pollination in a culturally appropriate manner.
- Enable and support ongoing costs of infrastructure, equipment, reagents and new staff, including provision of training and mentoring to new laboratory staff who have been engaged during the COVID-19 pandemic. Following strengthening ongoing capacity, laboratory services could be expanded to test for other pathogens.

### Strength and limitations

The use of FGDs with diverse stakeholders for Australia and the Region, with demonstrated expertise, skills and engagement helped identify health system and health security challenges and how to address them that are relevant to the Region. It allowed for participants to explore the context within which they are working and identify collective solutions by “piggybacking” on other participants [51].

Our study has some limitations. Firstly, the sample drawn from the study researchers’ networks may have led to a bias of experts that had ties to the Australian policy context. This meant at times the discussions centred on the role of funding inputs from the Australian government and other Australian based institutions. Secondly, the rapidly evolving situation of the COVID-19 pandemic and country responses meant that the expert opinions were based on the situation at a point in time. Any change in views as a result of recent changes in the dynamic of the pandemic and response activities could not be captured. Thirdly, as is the case with qualitative research, the findings from FGDs cannot be generalised directly to other settings outside of the countries in the Region, however the priority areas identified, such as localisation and strengthening surveillance for decision making, may resonate with other settings and warrant further investigation. Furthermore, country experts and participants were clear that these findings could not be generalised even for all countries within the Region but provide an expert opinion. In some FGDs, some participants’ opinions may be biased or incomplete due to the presence of other members in the discussion; however, all participants were given the option to share additional views, if any, with study researchers confidentially. Additionally, the findings reported here represent a collective view and not every individual perspective could be listed. Fourthly, the scope of our study was limited to the perspectives of health experts, which meant the role of sectors outside health were not considered. Finally, as content area experts, study researchers acknowledge the role of reflexivity in qualitative research (prior experiences, assumptions and beliefs) which may have influenced the research process [52].

## Conclusion

There is significant momentum across governments, NGOs, UN Agencies and Donors to embrace the integration of health system strengthening and health security concepts. However, there is little evidence of effective strategies for implementation on the ground. Our findings provide a starting point, to translate these ideas into priorities for implementation for the Pacific region. These include workforce development, risk communication, surveillance and laboratory capacity with localisation emerging as a cross cutting theme. Details on evidence-based strategies to tackle each of these individually were not examined, but our findings reinforced any approaches should be implemented with greater focus on local organisations. As the next phase of health system strengthening and health security planning are developed, evaluation of these initiatives will strengthen our knowledge on the value of integrating of health system strengthening and health security.

## Supporting information

S1 Checklist COREQ checklist. (PDF)

S1 File List of organisational affiliations of participants. (DOCX)

S2 File Focus group discussion participant guide. (DOCX)

S3 File Focus group discussion facilitator guide. (DOCX)

S4 File Summary of extracted themes and supporting quotes. (DOCX)
