# Supplementary material for: Expert perspectives on priorities for supporting health security in the Pacific region through health systems strengthening
Source: PLOS Glob Public Health. 2022 Sep 22;2(9):e0000529. doi: 10.1371/journal.pgph.0000529 (PMC10021329; doi:10.1371/journal.pgph.0000529)
Supplement: S1 File — (DOCX) [file pgph.0000529.s002.docx]

**S1 File. List of organisational affiliations of participants**

- Australian Global Health Alliance
- Australian Government Department of Foreign Affairs and Trade
- Australian Red Cross
- Burnet Institute
- CBM international
- College of Medicine, Nursing and Health Sciences in Suva, Fiji
- George Institute for Global Health
- Interplast Australia & New Zealand
- Marie Stopes international
- National Centre for Immunisation Research and Surveillance (NCIRS), Australia
- Pacific Community (SPC)
- Royal Australasian College of Surgeons (RACS)
- The Fred Hollows Foundation
- World Health Organization Country Office, Papua New Guinea
- World Vision Australia
