# Supplementary material for: Expert perspectives on priorities for supporting health security in the Pacific region through health systems strengthening
Source: PLOS Glob Public Health. 2022 Sep 22;2(9):e0000529. doi: 10.1371/journal.pgph.0000529 (PMC10021329; doi:10.1371/journal.pgph.0000529)
Supplement: S2 File — (DOCX) [file pgph.0000529.s003.docx]

**S2 File. Focus group discussion participant guide**

**Priorities for Health Security and Health Systems Strengthening**

**Focus Group Discussion**

**Participant guide**

The purpose of the focus group discussion is to examine the relationship between health security and health systems in the Indo-Pacific region and determine which aspects and specific initiatives should be prioritised for future investments in the short-medium term.

**Context**

The COVID-19 pandemic has highlighted the role of resilient health systems in health security and outbreak response. The World Health Organization (WHO) recently released a framework to drive renewed efforts for health system strengthening to improve health security, known as “Health Systems for Health Security” (HS for HS). Our discussion will be broadly considered in the context of this framework:


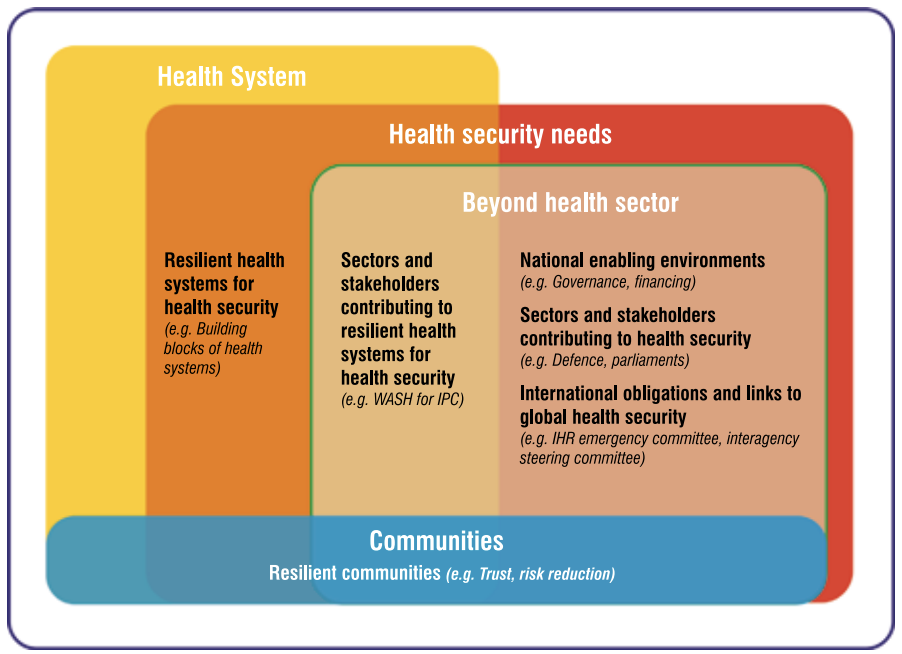


Source: World Health Organization, 2021, p. 7, Health systems for health security: a framework for developing capacities for International

Health Regulations, and components in health systems and other sectors that work in synergy to meet the demands imposed by health emergencies, <https://apps.who.int/iris/bitstream/handle/10665/342006/9789240029682-eng.pdf?sequence=1&isAllowed=y>.

The focus countries for our discussion are Timor Leste and Pacific Island Countries (PICs) which includes:

- Cook Islands
- Fiji
- Kiribati
- Republic of Marshall Islands
- Federated States of Micronesia
- Nauru
- Niue
- Palau
- Papua New Guinea
- Samoa
- Solomon Islands
- Tonga
- Tuvalu
- Vanuatu.

Health security will be defined as:

“*as the avoidance and containment of infectious disease threats with the potential to cause social and economic harms on a national, regional or global scale*” (Indo-Pacific Centre for Health Security, 2019, page ix, Health Security in the Indo-Pacific: State of the Region 2019).

As per the framework “Health Systems for Health Security is an approach that harmoniously brings together efforts to strengthen resources and  capacities  required for implementation of the International Health Regulations,  components in health  systems  and those in other sectors for effective management of health  emergencies, while maintaining the continuity of essential health services throughout.”

In this discussion, we will discuss key areas of health security that can directly benefit strengthening health systems for example Surveillance, Laboratory capacity, Public health workforce, Immunisation, Primary Health Care/Community Health, Risk Communication and community engagement, and Antimicrobial resistance.
