# Supplementary material for: Expert perspectives on priorities for supporting health security in the Pacific region through health systems strengthening
Source: PLOS Glob Public Health. 2022 Sep 22;2(9):e0000529. doi: 10.1371/journal.pgph.0000529 (PMC10021329; doi:10.1371/journal.pgph.0000529)
Supplement: S3 File — (DOCX) [file pgph.0000529.s004.docx]

**S3 File. Focus group discussion facilitator guide**

**Priorities for Health Security and Health Systems Strengthening**

**Focus Group Discussion**

**Facilitator Guide**

**Introduction**

Thank you all for coming.

My name is Dr Meru Sheel, I am an epidemiologist at the ANU. Along with me is Ms Nicole Rendell, who is working with me on this project.

The focus of today’s discussion is to examine the relationship between health security and health systems. We are particularly interested in your views on the key health system challenges facing countries in the Indo-Pacific region, that impact health security – and the Region’s ability to detect, avert and respond. Through this process we want to identify what initiatives should be prioritised to mitigate or avoid these challenges or strengthen health systems structures to improve health security initiatives in the region.

Along with Participant Information Sheet, you will have already received a list of our focus countries and working definitions to guide our discussion.

As per the WHO framework “Health Systems for Health Security is an approach that harmoniously brings together efforts to strengthen resources and capacities required for implementation of the International Health Regulations, components in health systems and those in other sectors for effective management of health emergencies, while maintaining the continuity of essential health services throughout.”

Today’s session will be recorded and the discussion will be documented through notetaking. These notes will then be reviewed and analysed and incorporated into a final report to identify the key priorities for health security in the region. The final report to be submitted to Australian Council for International Development (ACFID). No individuals or identifying information of individuals will be named in the report. You may choose to switch off your video if you prefer.

**PRESS RECORD**

You will have been sent an information sheet outlining information about your involvement in this discussion. Your participation is voluntary and you can withdraw at any time during the session, without negative consequences, however your contribution up until withdrawal will be retained. Have each of you read the information sheet and agree to participate, noting that the group discussion is being recorded and de-identified quotes from this session may be included as part of reporting?

- Yes from each participant – Continue
- No – Pause discussion to resolve concerns. The participant will again have the choice to leave the discussion by disconnecting from the meeting.

**Brief introductions of participants** – Please introduce yourself by providing your name, role, organisation and years of experience in health security and/or health systems.

**BEGIN DISCUSSION**

**Guiding questions**

1. Based on your experience, from both before and after the COVID-19 pandemic, what do you perceive as the key challenges facing health systems in the Pacific, in terms of health security?

- Have these challenges been exacerbated because of the pandemic, remained the same, or gotten better with the influx of COVID-19 investments?

o Consider:

▪ Surveillance

▪ Laboratory capacity

▪ Health Workforce

▪ Immunisation

▪ Primary Health Care/Community Health

▪ Risk Communication

▪ Antimicrobial resistance.

2. What could be done to strengthen health systems to ***avoid*** these challenges?

- Consider:

o Existing resources

o Health security needs within health systems

o Health security needs beyond the formal health system (eg community health, animal health, environmental health, water, sanitation and hygiene for infection prevention and control).

3. What could be done to strengthen health systems to ***mitigate*** these challenges?

- Consider:

o Existing resources

o Health security needs within health systems

- Health security needs beyond the formal health system (eg community health, animal health, environmental health, water, sanitation and hygiene for infection prevention and control).

4. In contrast, what do you perceive as the key strengths facing health systems in the Pacific, in terms of health security?

- How can these be enhanced or mobilised to manage or avoid the stated challenges?

5. What do you consider the top priority for strengthening regional health systems for improved health security?

- Where do you see the role of gender and disability inclusion in these priorities?

6. In terms of implementation, who is best placed to implement [prioritised investment identified from discussion] - an NGO, commercial or multilateral organisation?

• Please identify which kind of partner and why
