# Supplementary material for: Expert perspectives on priorities for supporting health security in the Pacific region through health systems strengthening
Source: PLOS Glob Public Health. 2022 Sep 22;2(9):e0000529. doi: 10.1371/journal.pgph.0000529 (PMC10021329; doi:10.1371/journal.pgph.0000529)
Supplement: S4 File — (DOCX) [file pgph.0000529.s005.docx]

**S4 File. Summary of extracted themes and supporting quotes**

| Extracted Theme | Supporting Quotes | |
| --- | --- | --- |
| Workforce development | - *“Workforce capacity building is constantly neglected in the Pacific. We know from the research and the data that there just isn’t enough population or people to be able to get the numbers that we need in terms of health care professionals.”* FGD#2 - “… *the workforce capacity is not sufficient for day-to-day operations let alone surge capacity…* *Need to address support for existing health professionals*” FGD#2 - *“…major opportunity was primary health care strengthening and a recognition that most frontline care does not require physicians”* FGD#2 - *“The concept of task shifting, building up capacity at the community level, that’s where we’ve seen success.”* FGD#2 - *“The more eager and hyper segmented our assistance becomes, the more it runs counter to sensible health workforce development…From a health security perspective and what workforce means, it actually means a generalised health care workforce with some public health capacities.”* FGD#4 - “*Countries like Australia have interest in fly in style but should never replace national rapid response. Sometimes necessary where there’s an acute surge. Coupled with long term capacity building*.” FGD#3 - “*We already had the view pre pandemic that there was a case for deepening the pool and broadening the pool of deployable public health expertise*.” FGD#4 - *“What we’ve learnt is there’s a need to invest in online facilities, in terms of primary health care training or advanced surgical training for experienced practitioners…* *there's a real need to look at ways [to]… invests in that sort of online capacity. Infrastructure training, modules, digital platforms.”* FGD#2 | |
| Risk communication | - “*This extends beyond the general public to the communication of advice that’s being given to government including the level of urgency applied to that advice*.” FGD#1 - “*Risk communication is an important part of any response. Just communication resources that have been produced elsewhere are rarely appropriate for the current country. Need capacity in place to identify need to rapidly adapt. They can then have resources made locally appropriate, field tested and rolled out.”* FGD#3 - *“Restoring funding to the ABC [Australian Broadcasting Corporation] Pacific Australia network…There are many communities where radio is still the lifeline to the outside world. Having access to that network again, they would be able to hear very informed, clear public health information.”* FGD#1 - “*Facebook [is the] only source of information in PNG [Papua New Guinea] at the moment and it is full of rumour and misinformation. Our programs are on Facebook trying to provide information but not using artificial intelligence. It’s very challenging to counter misinformation*.” FGD#1 | |
| Public health surveillance | - “*There was a lost opportunity or intent in the capacity and capability for research in line with the pandemic, and the crisis going on to give us real time data that is good for policy and decision-making*” FGD#3 - “*There’s information that gets gathered but countries being able to really make use of that and use it to drive their priorities. It’s a long term project*.” FGD#4 - “*It all starts with data, so having that disaggregated disability, gender, age, disaggregated data in the very beginning and how much that could help the response because responses are so urgent and immediate.*”FGD#1 - “*Only now have time [to] consider [the] pattern of distribution*.“ FGD#4 - *“One of the key elements to decentralisation is health records and access to them. There are some e-records at the central level but [but mostly] still using paper at community level. Need to move online as much as possible but need to ensure access at community level [which is] very difficult.” FGD#2* - *“We need to support identification of local champions for advocacy research. These are the people that can work with the data. Identify operational research priorities… They tend to work in public health but not in government. Senior and central. Tends to be academics at mid-level institutions. These champions can promote critical thinking and drive local responses [as] trusted technical partners*.” FGD#2 - “*Teams will come in with surveillance tools which can be great for a short time, but they’re not really building national capacity, so having the tools and the people trained on those tools ahead of time is very important to be able to exercise those capacities with tabletop exercises, simulations, and having the people not just from health*.” FGD#3 | |
| Laboratory capacity | - “*Lab strengthening … neglected for a long while*.” FGD#4 - “*Laboratory systems have improved ahead of pace but covering the cost of consumables is an ongoing factor*.” FGD#3 - “*One of the key issues with the emergency response was introducing new things rather improving existing things. For example, lab capacity was improved with surge in supplies of GeneXpert machines to support COVID-19 testing, but the systems in place have not been able to budget for their ongoing costs*.” FGD#3 | |
| Localisation | - “*Tailoring response and development strategies to countries – usually one size fits all applied to Pacific but each country has its own specific context that we need to take into account so it is better to tailor.” FGD#1* - *“Local empowerment… Institutional linkages are one [of] the most effective mechanisms… Partnerships between academic institutions are peripheral, NGOs vary but it’s not them either, it’s community organisations.” FGD#4* - *“Depends on issues – sometimes need more complex relationship. Much more nuanced. As local as possible in first instance but may need tailored support depending on what you’re looking at.” FGD#1* - *“We need to consider who are the critical stakeholders in the Pacific. Our organisation has had success collaborating with faith leaders. This approach uses what’s already trusted faith networks and civil society.” FGD#1* - *“There is a problem with over-piloting. Attempts to scale tend to fail because they are not developed with [the] right partners from the outset.” FGD#2* | |
| Other emergent topics | Disruption in routine immunisation during the COVID‑19 pandemic highlighted difficulties in maintaining progress of immunisation programs other than for COVID‑19. | - “Difficulties have emerged for the broader expanded program on immunisation. While we see a rapid acceleration of our first and then second dose coverage [of COVID‑19 vaccines] across the Pacific, data shows routine immunisation coverage, measles and rubella coverage for example, may have slipped as low as 50% coverage in a number of countries.” FGD#3 |
|  | Wellbeing of populations and the health workforce was negatively impacted by COVID‑19 measures. | - “Generally wellbeing is not addressed well in the health security preparations when the pandemic is quite lengthy. For example, health workforce and general population through lockdowns.” FGD#3 |
|  | Maintaining primary health care and community services during crises is vital but difficult. | - “Evidence shows more people died from maternal causes than Ebola [2014 outbreak in West Africa]. When there is a crisis, we should be allocating funding to what’s causing the most death and disability accordingly rather than focusing on what’s the ‘new thing’.”FGD#1 |
|  | Contribution of and learnings on infection prevention and control measures in the Pacific. | - “So if this pandemic was a measure of how good a system was intended, IPC [infection prevention and control], it exposed the system and we really need to do a lot of work in this area.” FGD#3 |
|  | Gaps in data on gender and people with disability. | - *“Both the Australian Humanitarian Partnership and Pacific Disaster Ready, set up in PNG [Papua New Guinea], Solomon Islands, Fiji and Timor Leste, are examples that have improved capacity among local disability organisations to advocate for disability inclusion. In terms of COVID‑19, this means more people at the table in planning the response [that consider disability inclusion].”* FGD#1 |
